# NO_2_ Gas Sensor Based on WO_3_/SiNWs Composite Structure

**DOI:** 10.3390/mi17020211

**Published:** 2026-02-05

**Authors:** Fengyun Sun, Encheng Zhang

**Affiliations:** 1 Engineering College, China University of Petroleum-Beijing at Karamay, Karamay 834000, China; 2 Heilongjiang Provincial Key Laboratory of Micro-Nano Sensitive Devices and Systems, Heilongjiang University, Harbin 150080, China; encheng.zhang@zymec.cn

**Keywords:** WO_3_, SiNWs, heterojunction, NO_2_ gas sensing

## Abstract

Although tungsten oxide (WO_3_)-based NO_2_ sensors have been extensively studied, achieving high sensitivity at low operating temperatures remains a significant challenge. To address this limitation, we designed a WO_3_/SiNWs heterojunction-based sensor, fabricated through metal-assisted chemical etching followed by hydrothermal synthesis. Structural and morphological analyses confirm the uniform integration of WO_3_ nanorods onto SiNWs and the establishment of an effective p–n junction. The optimized sensor exhibits a response of 238 toward 1 ppm NO_2_ at 127 °C with a response/recovery times of 14.8 s/99.2 s. The improved performance stems from the heterojunction-driven enhancement of charge carrier separation and surface adsorption sites, offering a viable route for developing low-power, high-performance gas sensors.

## 1. Introduction

Nitrogen dioxide (NO_2_) is a hazardous atmospheric pollutant that poses significant risks to human respiratory health and contributes to the formation of tropospheric ozone. Developing reliable, low-power gas sensors capable of sensitive and selective NO_2_ detection is therefore critical for environmental monitoring and public health protection [1]. Many gas sensors based on ZnO [2], WO_3_ [3], SnO_2_ [4], NiO [5], CuO [6,7], In_2_O_3_ [8], CeO_2_ [9], Fe_2_O_3_ [10], and InSe [11,12] have been extensively investigated for NO_2_ detection. Among various sensing materials, tungsten trioxide (WO_3_) not only serves as an effective additive to enhance the performance of various functional materials [13,14], but also demonstrates considerable potential for gas sensing applications [15,16].

However, conventional WO_3_-based sensors generally require operating temperatures above 200 °C to achieve sufficient response, leading to high energy consumption and limiting their practicality in portable scenarios [17]. Considerable efforts have been made to improve the sensing performance of WO_3_, including nanostructuring, doping, and heterojunction engineering [18,19,20]. In particular, constructing heterojunctions with silicon nanowires (SiNWs) represents a promising strategy to lower operating temperature while enhancing sensitivity, given the high surface-to-volume ratio and tunable electrical properties of SiNWs [21,22,23].

Recent studies have demonstrated the feasibility of integrating metal oxide with SiNWs. For example, Qin et al. [24] fabricated a composite array consisting of SiNWs and WO_3_ nanowires, which exhibited improved NO_2_ response at room temperature. Huang et al. [25] developed a Cu_2_O/SiNWs hybrid structure for ozone sensing, highlighting the benefit of such heterojunctions in gas detection. Despite these advances, the systematic understanding of interfacial charge transfer, morphological influence, and the sensing mechanism in WO_3_/SiNWs systems remains incomplete. Specifically, how the geometry of WO_3_ nanostructures, the quality of the heterojunction, and the resulting electronic interaction affect the overall NO_2_ sensing behavior have not been thoroughly elucidated.

In this work, we design and fabricate a WO_3_/SiNWs composite sensor through a two-step process involving metal-assisted chemical etching of SiNWs followed by hydrothermal growth of WO_3_ nanorods. We systematically investigate the relationship between material structure and sensing properties, with emphasis on the heterojunction-induced modulation of carrier transport and surface adsorption. Our results show that the optimized WO_3_/SiNWs sensor achieves significantly enhanced NO_2_ response at a reduced operating temperature of 127 °C, along with improved selectivity and a low detection limit. This study not only provides a feasible pathway toward high-performance, low-temperature NO_2_ sensors but also offers deeper insights into the heterojunction engineering of metal oxide–semiconductor nanocomposites for gas sensing applications.

## 2. Materials and Methods

### 2.1. Fabrication of WO_3_/SiNWs Composite Gas Sensors

P-type silicon wafers (oriented: <100>, resistivity: 0.001–0.005 Ω·cm) were employed as substrates. These wafers, with a diameter of 100 ± 0.2 mm and thickness of 525 ± 25 μm and featuring single-side polished surfaces, were diced into rectangular strips measuring 25 mm × 5 mm. Prior to etching, the silicon strips underwent a rigorous cleaning procedure to remove surface contaminants such as dust, organic residues, and native oxide layers.

**Figure 1:**
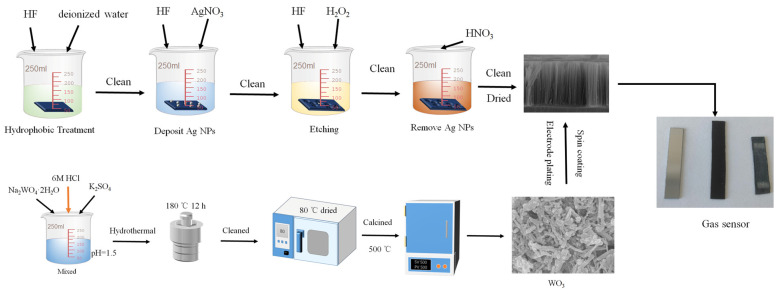
Preparation process of the WO_3_/SiNWs composite gas sensors.

Figure 1 shows the preparation process of the WO_3_/SiNWs composite gas sensors. A two-step metal-assisted chemical etching (MACE) process was used to fabricate silicon nanowires (SiNWs). The cleaned silicon substrates were first rendered hydrophobic by immersion in a solution of 10 mL HF (40 wt%) and 10 mL deionized water for 60 s, followed by thorough rinsing with deionized water. A deposition solution was then prepared by mixing appropriate volumes of HF (40 wt%), AgNO_3_ solution (0.025 M), and deionized water to achieve final concentrations of 4.4 M HF and 0.01 M AgNO_3_. The hydrophobized silicon substrates were immersed in this solution for 60 s with their polished surfaces facing upward to facilitate silver nanoparticle deposition. After rinsing, the substrates were transferred to an etching solution containing 4.4 M HF and 0.8 M H_2_O_2_, where etching was carried out for 1 h with visible bubble evolution observed on the silicon surface. Finally, the etched substrates were immersed in 68% HNO_3_ for 60 s to remove residual silver nanoparticles, thoroughly rinsed with deionized water, and dried on filter paper.

WO_3_ nanorods were synthesized via a hydrothermal method, in which the concentration of K_2_SO_4_ was systematically varied. In a representative procedure, 0.008 mol of Na_2_WO_4_·2H_2_O was dissolved in 50 mL of deionized water under stirring at 300 rpm. K_2_SO_4_ were added at molar ratios of 200% relative to the Na_2_WO_4_·2H_2_O precursor. The pH of the solution was then adjusted to 1.5 by dropwise addition of 6 M HCl under vigorous stirring at 800 rpm for 15 min. The mixture was transferred into a Teflon-lined autoclave and hydrothermally treated at 180 °C for 12 h. The resulting precipitates were collected, washed repeatedly three times with deionized water and ethanol via centrifugation, and then dried at 80 °C. The obtained powders were ground finely and calcined in a muffle furnace at 500 °C for 2 h with a heating rate of 5 °C/min.

The WO_3/_SiNWs composite sensors were fabricated by depositing hydrothermally synthesized WO_3_ nanorods onto the pre-etched SiNWs substrates. A homogeneous suspension was prepared by dispersing 0.2 g of WO_3_ powder in 4 mL of anhydrous ethanol, yielding a concentration of 50 mg/mL. This suspension was uniformly deposited onto the SiNWs substrates through five spin-coating cycles at 1500 rpm. Finally, two rectangular aluminum electrodes (4 mm × 2 mm, spaced 12 mm apart, with ~2 μm thickness) were thermally evaporated onto the composite surface under vacuum to complete the sensor assembly.

### 2.2. Materials Characterization

The crystalline structures of the synthesized materials were analyzed using X-ray diffraction (XRD, D8 Advance, Bruker,  Karlsruhe, Germany) with Cu Kα radiation. Morphological features were examined by scanning electron microscopy (SEM, S-3400, Hitachi, Tokyo, Japan) and transmission electron microscopy (TEM, Talos F200X G2, Thermo Fisher Scientific, Waltham, MA, USA). Surface chemical composition and elemental oxidation states were determined by X-ray photoelectron spectroscopy (XPS, Escalab 250Xi, Thermo Fisher Scientific, Waltham, MA, USA) with Al Kα radiation.

**Figure 2:**
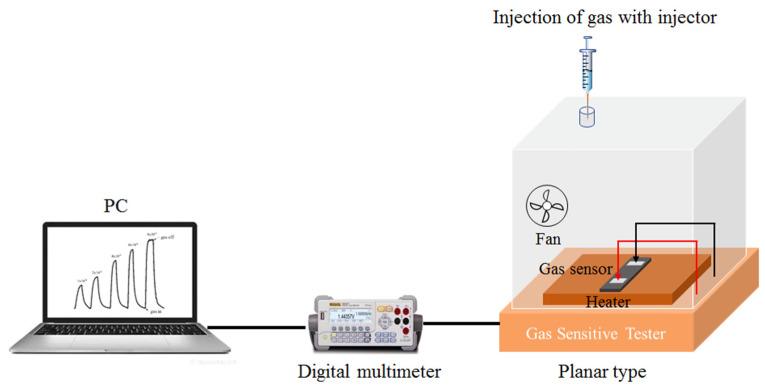
Gas sensitive tester.

The NO_2_ gas sensing performance of the WO_3_/SiNWs composite was evaluated with a static gas distribution system at 36% RH (the ambient relative humidity), as shown in Figure 2. In this study, the NO_2_ gas sensing response is defined as *R*_a_/*R*_g_, where *R*_a_ and *R*_g_ denote the steady-state resistance of the sensor in air and in the target gas atmosphere, respectively.

## 3. Results

### 3.1. Structural and Morphological Characteristics of WO_3_/SiNWs Composite

**Figure 3:**
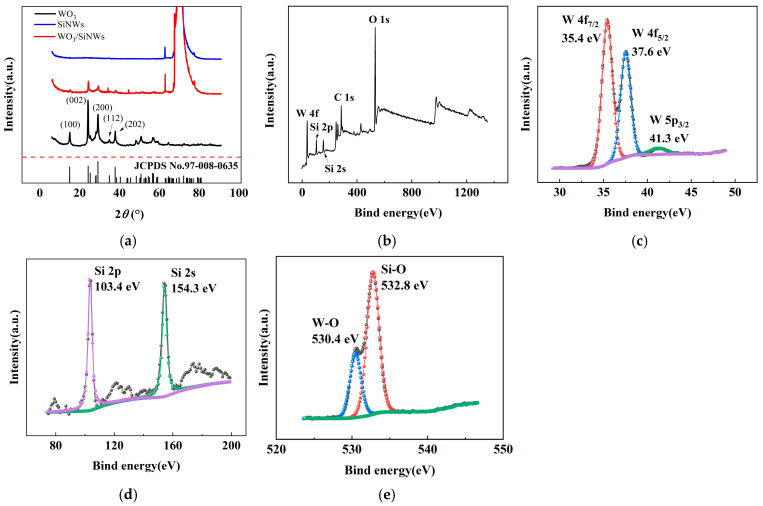
XRD and XPS pattern of the WO_3_/SiNWs heterostructure: (**a**) XRD pattern; (**b**) XPS survey spectrum, and high-resolution spectra of (**c**) W 4f, (**d**) Si 2p/2s, and (**e**) O 1s regions for the WO_3_/SiNWs composite.

The crystalline structures of pristine WO_3_, SiNWs, and the WO_3_/SiNWs composite were examined by XRD, as shown in Figure 3a. The diffraction pattern of WO_3_ displays characteristic peaks at 2θ = 13.98°, 23.13°, 28.17°, 33.81°, and 36.81°, which are assigned to the (100), (002), (200), (112), and (202) planes of hexagonal WO_3_ (JCPDS No. 91-008-0635). For SiNWs, distinct peaks are observed at 2θ = 61.87° and 69.22°, corresponding to the crystalline silicon phase. In the composite, both sets of peaks are retained, confirming the coexistence of WO_3_ and Si. The lower relative intensity of the WO_3_ peaks can be attributed to its lower mass loading in the hybrid, indicating effective hybridization without destruction of the crystalline frameworks of either component.

XPS analysis (Figure 3b–e) was conducted to probe the surface chemistry and bonding states. The survey spectrum in Figure 3b clearly shows peaks of W, O, and Si, in agreement with the expected composite composition. In the high-resolution W 4f region (Figure 3c), two prominent peaks appear at 35.4 eV and 37.6 eV, assigned to the W 4f_7_/_2_ and W 4f_5_/_2_ states of W^6+^, respectively. The peak at 41.3 eV corresponds to W 5p_3_/_2_. The Si 2p and Si 2s peaks are located at 103.4 eV and 154.3 eV (Figure 3d), confirming silicon in a predominantly oxidized environment. Deconvolution of the O 1s spectrum (Figure 3e) yields two contributions: the component at 530.4 eV arises from lattice oxygen in W–O bonds, whereas the peak at 532.8 eV is associated with oxygen species in Si–O, likely due to surface oxidation of SiNWs.

**Figure 4:**
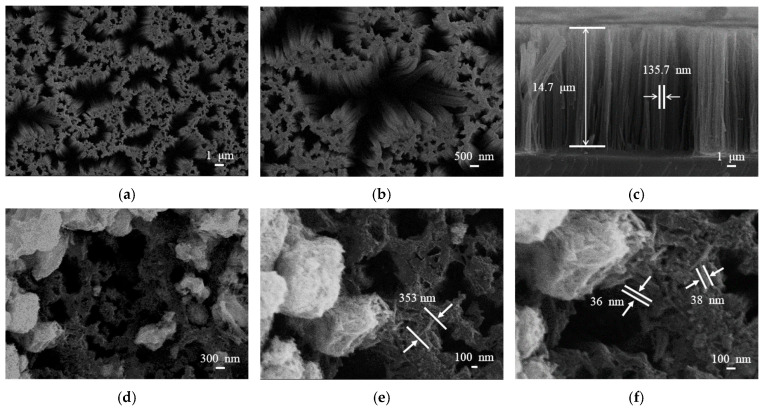
SEM images of (**a**–**c**) bare SiNWs and (**d**–**f**) WO_3_/SiNWs composite: (**a**) plan view (5000×), (**b**) detailed nanowire morphology (10,000×), (**c**) cross-sectional view, (**d**) composite surface (15,000×), (**e**) WO_3_ nanorod distribution (30,000×), and (**f**) interfacial structure (45,000×).

SEM images in Figure 4 provide morphological details of bare SiNWs and the WO_3_/SiNWs composite. The bare SiNWs (Figure 4a–c) present a vertically aligned porous network with smooth surfaces. Statistical analysis indicates an average length of ~14 μm and diameter of ~136 nm. After hydrothermal growth of WO_3_, the composite (Figure 4d–f) shows uniform coverage of the SiNWs scaffold by WO_3_ nanorods, which have an average length of ~350 nm and diameter of 35–40 nm. Notably, a fraction of the nanorods remain embedded within the porous SiNWs matrix, suggesting strong interfacial bonding between the two phases.

**Figure 5:**
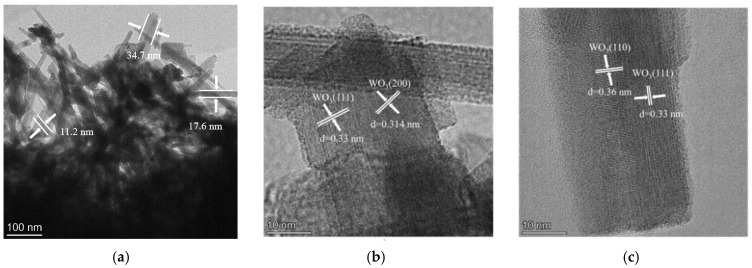

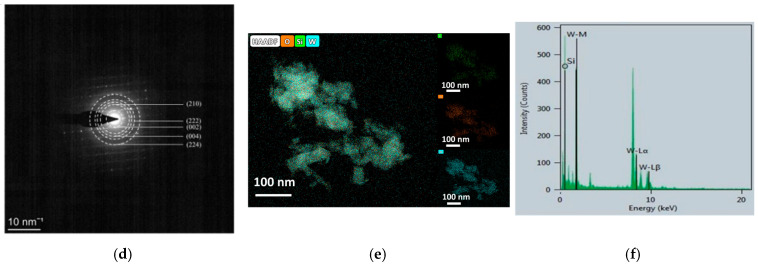
TEM analysis of WO_3_/SiNWs: (**a**) low-magnification overview, (**b**,**c**) high-resolution images at 1,050,000× showing lattice fringes and (**d**) corresponding SAED pattern, (**e**) elemental analysis of HAADF-STEM image and (**f**) EDS spectrum.

To gain deeper insight into the microstructure and crystal orientation, TEM and associated analyses were performed (Figure 5). Figure 5a shows stacked nanorods with average dimensions of ~350 nm (length) and 11~35 nm (width). High-resolution TEM images (Figure 5b,c) reveal clear lattice fringes with measured spacings of 0.33 nm, 0.314 nm, and 0.36 nm, which match the (111), (200), and (110) planes of hexagonal WO_3_, respectively. These observations confirm that the WO_3_ nanorods grow with well-defined crystallographic orientation and remain structurally coherent. The SAED pattern in Figure 5d exhibits concentric rings composed of sharp diffraction spots. The discrete spots are attributed to single-crystalline silicon, whereas the continuous rings correspond to polycrystalline WO_3_ and can be indexed to the (210), (222), (002), (004), and (224) planes of hexagonal WO_3_, consistent with XRD results. This diffraction signature collectively confirms the coexistence of highly crystalline Si and WO_3_ phases with distinct structural ordering.

Elemental distribution and chemical purity were assessed by EDS mapping and point analysis (Figure 5e,f). The elemental maps demonstrate homogeneous spatial distribution of Si, O, and W across the imaged region. The corresponding EDS spectrum (Figure 5f) displays prominent signals from these three elements with no detectable impurity peaks, confirming the high chemical purity of the composite. Together, these results provide compelling evidence that the WO_3_/SiNWs composite possesses well-defined crystallographic orientation, phase purity, and intimate interfacial contact—key characteristics that underpin its enhanced sensing functionality.

### 3.2. NO_2_ Sensing Performance Evaluation

**Figure 6:**
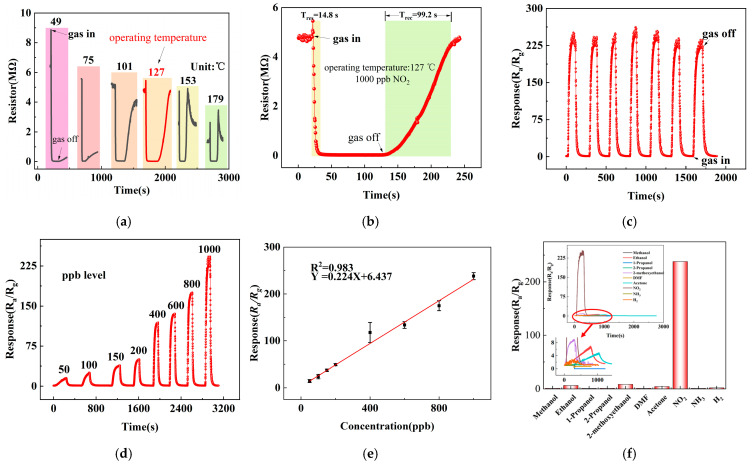
NO_2_ sensing performance: (**a**) temperature-dependent response to 1 ppm NO_2_, (**b**) dynamic response/recovery curve at 127 °C, (**c**) repeatability test over seven cycles, (**d**) response to different NO_2_ concentrations, (**e**) linear fitting of concentration-response relationship, and (**f**) selectivity toward various gases.

Figure 6a illustrates the sensor response to 1 ppm NO_2_ at different operating temperatures. At relatively low temperatures (49 °C, 75 °C, and 101 °C), the sensor exhibited incomplete recovery due to insufficient desorption kinetics. At 153 °C, the sensor exhibited an unstable resistance response when exposed to 1 ppm NO_2_, characterized by a sharp initial increase followed by an abrupt decrease. Upon switching back to the baseline atmosphere, the resistance rose rapidly and temporarily exceeded the baseline value with notable instability. Prolonged recovery time was required before the resistance returned to the baseline level. The sensitivity under these conditions was measured to be 117. An optimal operating temperature of 127 °C was identified, where a balance between adsorption and desorption was achieved, yielding a maximum response of 238 toward 1 ppm NO_2_. The corresponding dynamic response curve at this temperature (Figure 6b) shows response and recovery times of 14.8 s and 99.2 s, respectively.

Repeatability was further investigated through seven consecutive exposures to 1 ppm NO_2_ (Figure 6c). The response values remained highly consistent (232, 227, 233, 244, 236, 226, and 221), with a relative standard deviation (RSD) of 3.25%, confirming excellent operational stability and reproducibility.

The concentration-dependent response is shown in Figure 6d. The sensor was exposed to NO_2_ concentrations ranging from 50 to 1000 ppb, yielding responses of 14.5, 24.1, 37.4, 49, 117.7, 133.8, and 238, respectively. A strong linear relationship between response and NO_2_ concentration was observed (Figure 6e), with a fitting coefficient R^2^ = 0.983 and a sensitivity of 0.224 per ppb. The theoretical detection limit was estimated through noise analysis based on 60 consecutive baseline measurements, giving a standard deviation of 0.000040683 and a root mean square noise of 0.000823443.

Selectivity was assessed to various interfering gases at 100 ppm (methanol, ethanol, n-propanol, isopropanol, ethylene glycol monomethyl ether, DMF, acetone, NH_3_, H_2_) along with 1 ppm NO_2_. As depicted in Figure 6f, while minor cross-sensitivity was observed toward acetone and ethylene glycol monomethyl ether, the sensor displayed pronounced selectivity to NO_2_. These results demonstrate superior performance in comparison with recently reported WO_3_-based gas sensors, as summarized in Table 1.

The work reported in this paper.

### 3.3. Gas Sensing Mechanism of WO_3_/SiNWs Heterostructure

**Figure 7:**
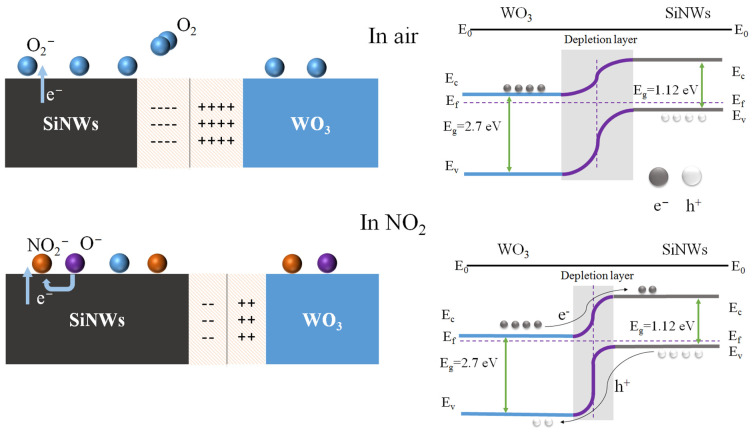
Schematic illustration of the gas sensing mechanism in WO_3_/SiNWs heterostructure.

The enhanced NO_2_ sensing mechanism of the WO_3_/SiNWs heterostructure is illustrated schematically in Figure 7. The formation of a p-n heterojunction at the interface between n-type WO_3_ (band gap ≈ 2.7 eV) and p-type SiNWs (band gap ≈ 1.12 eV) plays a critical role in modulating the sensing behavior. Upon contact, the difference in Fermi levels drives charge carrier redistribution across the interface: holes diffuse from p-SiNWs to n-WO_3_, while electrons transfer in the opposite direction. This carrier migration leads to band bending and establishes a space-charge region through electron–hole recombination near the junction interface. The resulting built-in electric field effectively suppresses further charge diffusion until the system reaches thermodynamic equilibrium.

Upon exposure to air, oxygen molecules adsorb onto the WO_3_ surface and extract electrons from the conduction band, forming ionosorbed oxygen species. When NO_2_ is introduced, its strong electron-withdrawing capability enables further electron extraction from the composite, accompanied by hole injection into the valence band, as represented by the following surface reaction [2]:

(1) O_2_(g) → O_2_(ads)

(2) O_2_(ads) + e^−^ → O_2_^−^(ads)

(3) NO_2_(gas) → NO_2_(ads)

(4) NO_2_(ads) + e^−^ → NO_2_^−^(ads)

(5) NO_2_(ads) + O_2_^−^(ads) + 2e^−^ → NO_2_^−^(ads) + 2O^−^(ads)

(6) NO_2_^−^(ads) + 2O^−^(ads) + e^−^ → NO_2_(ads) + 2O^2−^(ads)

Upon exposure to oxidizing NO_2_ gas, pre-adsorbed oxygen species and NO_2_ molecules act synergistically to extract electrons, generating a high density of holes at the WO_3_ surface. When the accumulated hole concentration exceeds the intrinsic electron concentration, a localized electronic transition occurs in the surface region, converting it into a p-type-like behavior and forming an inversion layer where holes become the dominant charge carriers [30]. These induced holes can subsequently migrate together with intrinsic hole carriers from the p-type SiNWs substrate, thereby disrupting the equilibrium of the depletion region. As a result, the partial neutralization of diffused electrons from WO_3_ by holes supplied by the silicon nanowires allows more NO_2_ molecules to adsorb onto the WO_3_ surface, enhancing the gas-sensing response. Moreover, the isolated silicon nanowires are interconnected via WO_3_ nanorods, establishing additional conductive pathways beyond the dominant current path along the WO_3_ surface. This configuration enables more silicon nanowires to act as active sensing units, providing a larger effective sensing area and consequently further boosting the gas-sensing response of the composite sensor [24].

## 4. Conclusions

This study successfully fabricated a high-performance NO_2_ sensor based on a WO_3_/SiNWs heterostructure. Comprehensive structural and morphological characterization confirmed the uniform integration of hexagonal-phase WO_3_ nanorods onto a silicon nanowire substrate, forming a well-defined p–n heterojunction interface. The optimized sensor exhibited a response of 238 toward 1 ppm NO_2_ at a low operating temperature of 127 °C (lower than the 200 °C required for pure WO_3_), with response and recovery times of 14.8 s and 99.2 s, respectively. Furthermore, the device demonstrated good reproducibility. The enhanced sensing performance is attributed to band alignment and space-charge modulation at the WO_3_/SiNWs interface, which facilitates effective charge carrier separation and enhances NO_2_ adsorption. These findings indicate that the WO_3_/SiNWs heterostructure holds significant potential for the development of low-power, high-performance gas sensors for environmental monitoring.

## Figures and Tables

**Table 1:** NO_2_ gas sensing performance of the WO_3_-based gas sensor.

Materials	Methods	Morphology	Working Temperature (°C)	NO_2_ Concentration (ppm)	Response (*R*_g_/*R*_a_)	Response Time/Recovery Time (s)	Reference
Co-WO_3_	hydrothermal	nanoplates	200	10	207.76	15/23	[20]
WO_3_	acid precipitation	nanoplate	150	5	170	178/76	[26]
WO_3_	sol–gel	nanoparticles	200	100	34%	24/5	[27]
Zn mixed WO_3_	hydrothermal	nanoflowers	150	100	321.81	4/150	[28]
WO_3_	electrospinning	nanofibers	200	1	12.523	11/26	[29]
WO_3_/SiNWs	hydrothermal	nanorods	127	1	238	14.8/99.2	This work

## Data Availability

The data that support the findings of this study are available from the corresponding author upon reasonable request.
